# Novel QSAR Approach for a Regression Model of Clearance That Combines DeepSnap-Deep Learning and Conventional Machine Learning

**DOI:** 10.1021/acsomega.2c00261

**Published:** 2022-05-11

**Authors:** Hideaki Mamada, Yukihiro Nomura, Yoshihiro Uesawa

**Affiliations:** † Department of Medical Molecular Informatics, Meiji Pharmaceutical University, 2-522-1, Noshio, Kiyose, Tokyo 204-8588, Japan; ‡ Drug Metabolism and Pharmacokinetics Research Laboratories, Central Pharmaceutical Research Institute, Japan Tobacco Inc., 1-1, Murasaki-cho, Takatsuki, Osaka 569-1125, Japan

## Abstract

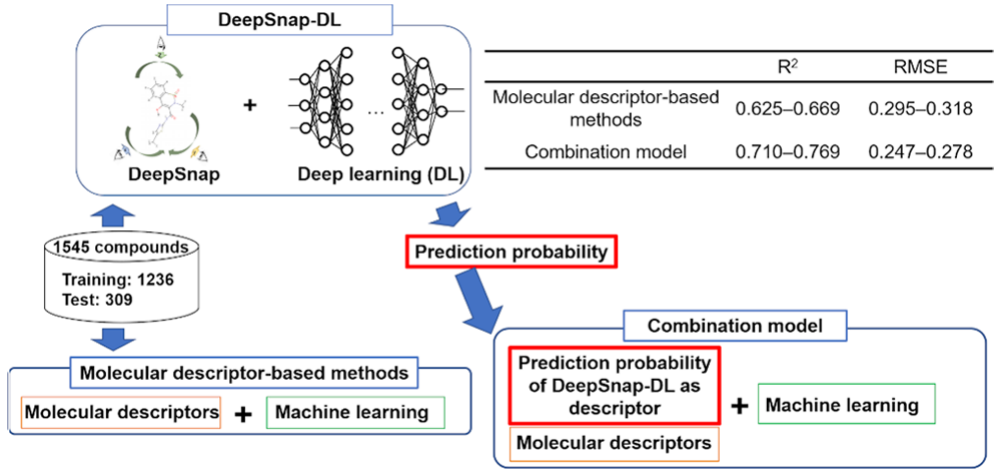

The toxicity, absorption, distribution, metabolism, and excretion properties of some targets are difficult to predict by quantitative structure–activity relationship analysis. Therefore, there is a need for a new prediction method that performs well for these targets. The aim of this study was to develop a new regression model of rat clearance (CL). We constructed a regression model using 1545 in-house compounds for which we had rat CL data. Molecular descriptors were calculated using molecular operating environment, alvaDesc, and ADMET Predictor software. The classification model of DeepSnap and Deep Learning (DeepSnap-DL) with images of the three-dimensional chemical structures of compounds as features was constructed, and the prediction probabilities for each compound were calculated. For molecular descriptor-based methods that use molecular descriptors and conventional machine learning algorithms selected by DataRobot, the correlation coefficient (*R*^2^) and root mean square error (RMSE) were 0.625–0.669 and 0.295–0.318, respectively. We combined molecular descriptors and prediction probability of DeepSnap-DL as features and developed a novel regression method we called the combination model. In the combination model with these two types of features and conventional algorithms selected by DataRobot, *R*^2^ and RMSE were 0.710–0.769 and 0.247–0.278, respectively. This finding shows that the combination model performed better than molecular descriptor-based methods. Our combination model will contribute to the design of more rational compounds for drug discovery. This method may be applicable not only to rat CL but also to other pharmacokinetic and pharmacological activity and toxicity parameters; therefore, applying it to other parameters may help to accelerate drug discovery.

## Introduction

The quantitative structure–activity relationship (QSAR) method uses structural features of compounds to predict their absorption, distribution, metabolism, excretion, and toxicity (ADMET) parameters virtually, which can reduce the time and effort required for their synthesis and subsequent experimental determination of their parameters.^1−3^ Hence, the QSAR method can increase the efficiency of drug discovery. Construction of QSAR prediction models is based mainly on molecular descriptors or fingerprints as features of compounds and conventional machine learning (ML) algorithms such as partial least squares regression, neural networks, random forest, support vector machines, and XGBoost.^4−9^ In this study, we defined conventional ML as a method that uses molecular descriptors and fingerprints as features and applies algorithms other than deep learning (DL) algorithms. Among these conventional ML algorithms, XGBoost has been used successfully in cheminformatics, and for some parameters, it produced results that were as good as those obtained using DL algorithms.^10^ Indeed, for tabular data, XGBoost outperformed DL algorithms.^11^

DL algorithms were developed primarily for image recognition and, in 2012, they attracted considerable attention when a team demonstrated their considerable prediction performance and won a large-scale competition for image recognition.^12^ Moreover, some DL prediction models have surpassed the image recognition ability of humans.^13^ The high performance of DL models in image recognition raised the possibility of applying these models to QSAR. Recently, we developed a DeepSnap and Deep Learning (DeepSnap-DL) method that applies DL using images of the three-dimensional chemical structures of compounds as features.^14−21^ Compared with conventional ML, DeepSnap-DL showed better predictive accuracy for several toxicity and pharmacokinetic parameters.^14−21^ We also developed a new classification model by combining molecular descriptor-based methods and DeepSnap-DL.^21^ When we applied this classification model to predict the rat clearance (CL), the area under the curve (AUC) (AUC = 0.943) was better than that obtained with a molecular descriptor-based method (AUC = 0.883).^21^ Although this method showed high performance, it was used in classification models, and its application to regression models is desired.

CL is an important pharmacokinetic parameter that is related to compounds’ exposure.^22^ In the early stages of drug discovery, rat CL is often used as an index guide to identify compounds that have an acceptable pharmacokinetic profile.^23^ However, to reduce cost, time, and animal usage, it is desirable to use a QSAR approach to predict rat CL before synthesizing and testing compounds. Previously, we reported a classification model for rat CL that showed high performance.^21^ However, at the drug discovery stage, the optimal CL may vary from project to project because of different target profiles, and therefore, the appropriate classification thresholds also may vary. For this reason, it is desirable to construct not only classification models but also regression models that have high performance. Kosugi and Hosea^24^ reported a regression model of rat CL that was constructed using conventional ML and 1114 of their in-house compounds. The prediction performance of this model was not sufficient [correlation coefficient (*R*^2^) = 0.555; root mean square error (RMSE) = 0.332]. Although rat CL prediction is an important evaluation target in drug discovery, it is difficult to predict by conventional ML. Therefore, in this study, we developed a new regression model that combined DeepSnap-DL and molecular descriptor-based methods for rat CL. Moreover, we also evaluated the applicability of this method to other parameters, which were reported as representative benchmark datasets.^25−27^

## Results

### Separation of Compounds into Five Different Datasets and Their Verification by Chemical Space Analysis for Rat CL

**Figure 1:**
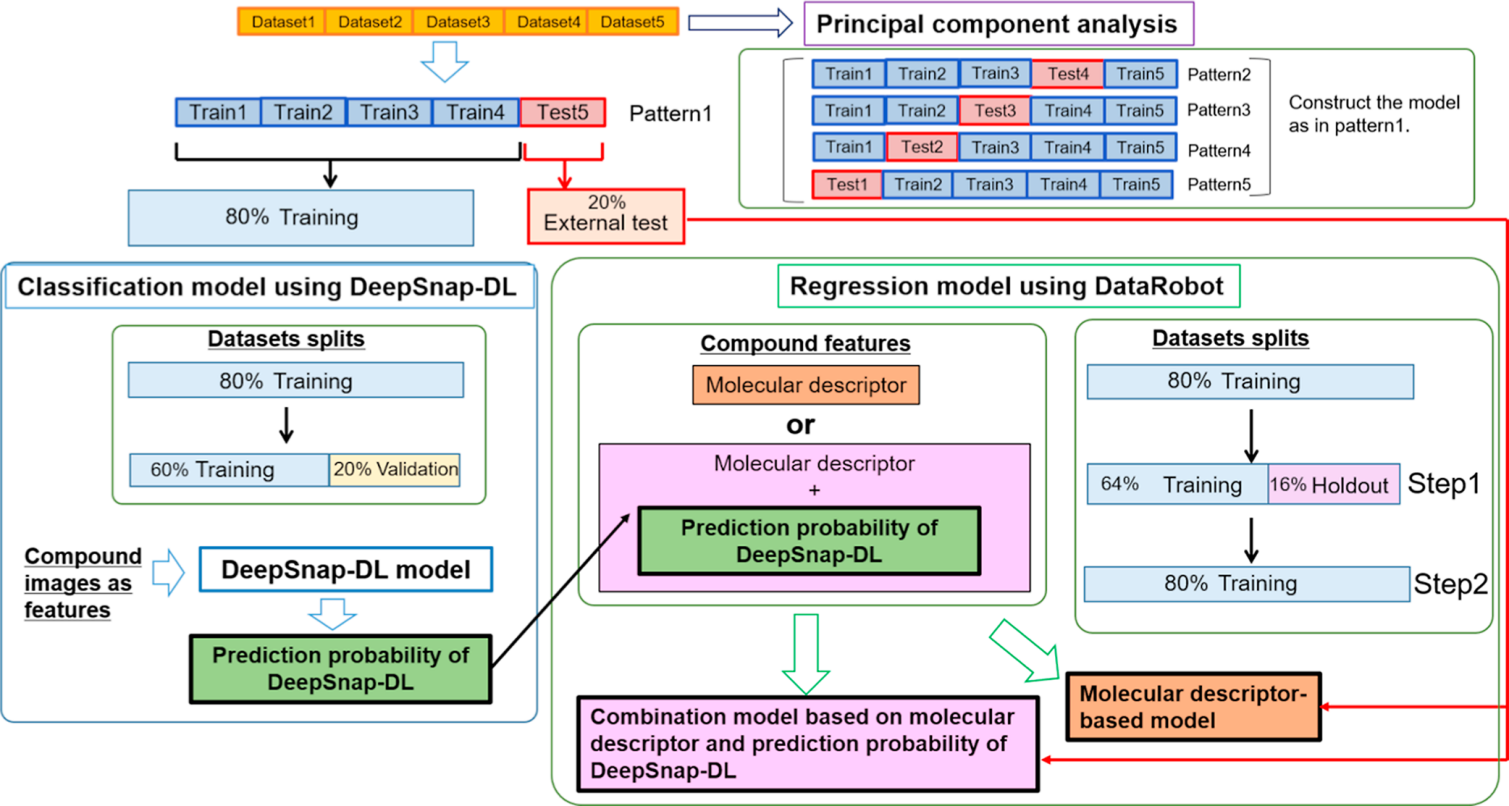
Flowchart of the modeling process for rat CL prediction.

**Figure 2:**
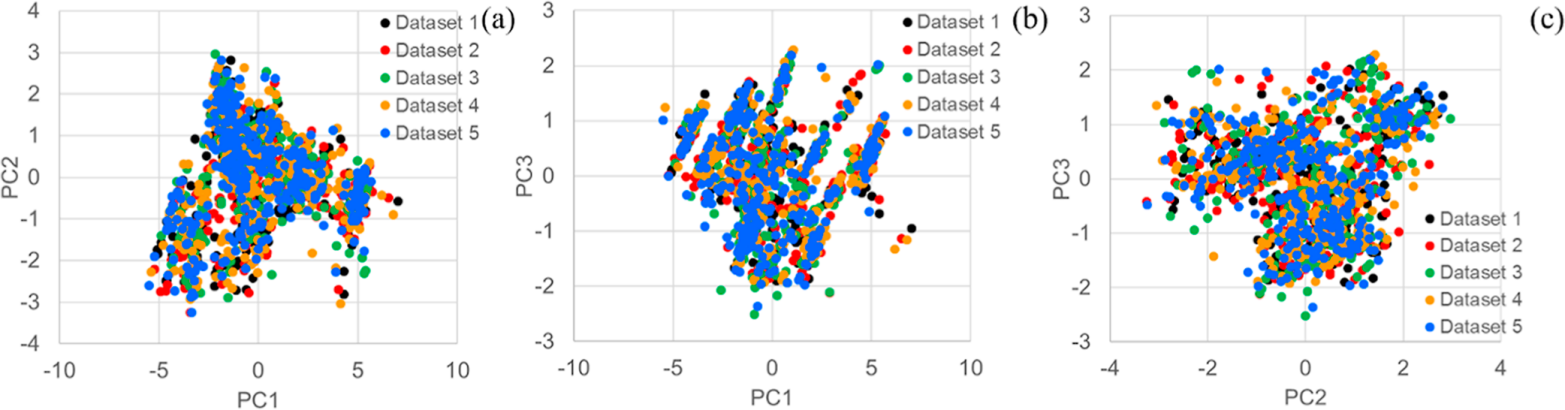
Chemical space distribution of five datasets by PCA using 11 representative molecular descriptors (*n* = 1545). (a) PC1 (62.3%) and PC2 (12.0%). (b) PC1 (62.3%) and PC3 (8.0%). (c) PC2 (12.0%) and PC3 (8.0%). Each dot represents a compound (*n* = 5 × 309). The 11 representative molecular descriptors were molecular weight, *S* log *P* (log octanol/water partition coefficient), topological polar surface area, *h*_log *D* (octanol/water distribution coefficient [pH 7]), *h*_p*K*_a_ (acidity [pH 7]), *h*_p*K*_b_ (basicity [pH 7]), a_acc (number of H-bond acceptor atoms), a_don (number of H-bond donor atoms), a_aro (number of aromatic atoms), b_ar (number of aromatic bonds), and b_rotN (number of rotatable bonds). The principal components were calculated from PC1 to PC3.

The rat CL dataset used in this study contained the same 1545 in-house compounds used in a previous study.^21^ After applying stratified random sampling, the compounds were separated randomly into five different datasets (Figure 1). Principal component analysis (PCA) was used to investigate the correctness of the compound separation using 11 representative molecular descriptors. PCA showed the distribution of the chemical space in the dataset as previously reported.^28^ The first three components, principal component (PC)1, PC2, and PC3, explained 62.3, 12.0, and 8.0% of the variance, respectively. The compounds were effectively separated into five different datasets as shown in Figure 2.

The five datasets were divided as 80% training and 20% test datasets on each pattern. The 80% training dataset was used by DeepSnap-DL to construct classification models using molecular images. For example, to construct classification models on pattern 1, the 80% training datasets (Train1, Train2, Train3, and Train4) were separated as 60% training and 20% validation datasets. Then, classification models were used to calculate the prediction probability of DeepSnap-DL. For regression models using DataRobot, the 80% training dataset was used to construct regression models using molecular descriptors (molecular descriptor-based methods) or a combination of the prediction probability of DeepSnap-DL and molecular descriptors (combination model). For example, on pattern 1, training datasets (Train1, Train2, Train3, and Train4) were separated as 64% training and 16% hold-out test datasets, and fivefold cross-validation was implemented using the 64% training datasets to choose molecular descriptors (step 1, Figure 1). The final models were constructed using all the training data (80%), and fivefold cross-validation was implemented. These models were used to calculate the evaluation metrics of the external test datasets (step 2, Figure 1).

### Molecular Descriptor-Based Method: Construction of the CL Prediction Model Using Molecular Descriptors Using DataRobot

Multiple regression models were constructed by DataRobot,^29,30^ an automated ML platform, using 4331–4337 molecular descriptors for each of the five dataset patterns (Figure 1). The RMSEs of internal validation for each of the five dataset patterns were calculated, and the algorithm that gave the lowest RMSE for each dataset was selected (Table 1; Table S1). On the basis of these results, we chose the single model or ensemble model of the conventional algorithm (Table S1). In the internal validations, *R*^2^ and RMSE were 0.573–0.595 and 0.328–0.338, and for the test dataset, *R*^2^ and RMSE were 0.625–0.669 and 0.295–0.318, respectively (Table 1).

**Table 1:** Internal Validation and External Test Results^a^

internal validation results	*R*^2^					RMSE				
methods	Pattern 1	Pattern 2	Pattern 3	Pattern 4	Pattern 5	Pattern 1	Pattern 2	Pattern 3	Pattern 4	Pattern 5
molecular descriptor-based methods	0.573	0.580	0.582	0.595	0.576	0.338	0.334	0.334	0.328	0.337
combination model	0.676	0.688	0.692	0.702	0.692	0.294	0.288	0.287	0.282	0.287

external test results	*R*^2^					RMSE				
methods	Pattern 1	Pattern 2	Pattern 3	Pattern 4	Pattern 5	Pattern 1	Pattern 2	Pattern 3	Pattern 4	Pattern 5
molecular descriptor-based methods	0.669	0.653	0.650	0.625	0.649	0.295	0.307	0.306	0.318	0.305
combination model	0.769	0.730	0.740	0.710	0.730	0.247	0.272	0.262	0.278	0.266

a The internal validation and test results were predicted by DataRobot using 4331–4338 descriptors for seed = 1. *R*^2^, correlation coefficient; RMSE, root mean square error. Combination model, combination of using prediction probability of DeepSnap and Deep Learning and molecular descriptors.

### Combination Model: Construction of the CL Prediction Model by Combining Molecular Descriptors and Prediction Probability of DeepSnap-DL Using DataRobot

Multiple regression models were constructed using 4332–4338 descriptors selected by DataRobot for the five dataset patterns (Figure 1). The RMSEs of internal validation for the five dataset patterns were calculated, and the algorithm that showed the lowest RMSE for each dataset pattern was selected (Table 1; Table S1). A single model of a conventional algorithm was chosen for each (Table S1). In the internal validation, *R*^2^ and RMSE were 0.676–0.702 and 0.282–0.294, and in the test set, *R*^2^ and RMSE were 0.710–0.769 and 0.247–0.278, respectively (Table 1).

### Feature Importance in the Prediction Models

The feature importance of the descriptors was calculated based on the permutation importance when the prediction model was constructed. The top 10 descriptors with the highest average effect in the prediction model using the molecular descriptor-based methods are listed in Table 2. The nFCharge+ and BCUT_SLOGP_0 descriptors, which are related to charge and lipophilicity of compounds, were the top ranked features of importance. The top 10 descriptors with the highest average effect in the prediction model using the combination model are listed in Table 3. Among these features, the prediction probability of DeepSnap-DL was the highest ranked, with an average effect of 1, followed by BCUT_SLOGP_0, which is related to lipophilicity, with an average effect of 0.107.

**Table 2:** Top 10 Molecular Descriptors in the Molecular Descriptor-Based Methods^a^

descriptor	software to calculate molecular descriptor	description	average effect
nFCharge+	MOE	number of atoms with a positive formal charge	0.513
BCUT_SLOGP_0	MOE	descriptors using atomic contribution to log *P*	0.496
MATS2p	alvaDesc	descriptor-related polarizability	0.418
B09[N–O]	alvaDesc	presence/absence of N–O bond at some topological distance	0.375
FCharge+	MOE	sum of the positive formal charges contained in a molecule	0.317
SMR_VSA3	MOE	descriptor-related molar refraction	0.311
FCharge+_max	MOE	largest number of positive formal charges contained in a molecule	0.297
h_pstrain	MOE	strain energy needed to convert all protonation states into the input protonation state	0.294
MATS6e	alvaDesc	descriptor-related electronegativity	0.242
F09[N–O]	alvaDesc	frequency of N–O bond at some topological distance	0.238

a The top 10 descriptors were calculated based on the permutation importance of the prediction models using the molecular descriptor-based methods for seed = 1. Average effect was calculated based on five different prediction models using Patterns 1–5 (Figure 1). MOE, molecular operating environment. Log *P*, log octanol/water partition coefficient.

**Table 3:** Top 10 Descriptors in the Molecular Descriptor and Prediction Probability of DeepSnap-DL for the Combination Model^a^

descriptor	software to calculate molecular descriptor	description	average effect
prediction probability of DeepSnap-DL		prediction probability calculated by DeepSnap-DL	1.000
BCUT_SLOGP_0	MOE	descriptors using atomic contribution to log *P*	0.107
SMR_VSA3	MOE	descriptor-related molar refraction	0.088
FCharge+_max	MOE	largest number of positive formal charges contained in a molecule	0.064
MATS2p	alvaDesc	descriptor-related polarizability	0.054
T_MIRyy	ADMET predictor	descriptor-related topological equivalent of MIRyy___3D (medium relative principal moment of inertia), but without mass weighting	0.050
FCharge+	MOE	sum of the positive formal charges contained in a molecule	0.049
nFCharge+	MOE	number of atoms with a positive formal charge	0.045
F09[N–O]	alvaDesc	frequency of the N–O bond at some topological distance	0.040
RDF020m	alvaDesc	descriptor-related radial distribution function weighted by mass	0.038

a The top 10 descriptors were calculated based on the permutation importance of the prediction models using the molecular descriptor and prediction probability of DeepSnap-DL for the combination model for seed = 1. The average effect was calculated based on five different prediction models using Patterns 1–5 (Figure 1). DeepSnap-DL, DeepSnap and Deep Learning; MOE, molecular operating environment. Log *P*, log octanol/water partition coefficient.

## Discussion

We developed a classification model with high prediction performance by combining DeepSnap-DL and molecular descriptor-based methods for rat CL prediction.^21^ However, for drug discovery, it is desirable to apply a regression model because the optimal compound profile differs depending on the projects. Therefore, we developed a new regression model with high performance by combining DeepSnap-DL and molecular descriptor-based methods.

In this study, to avoid a chance result, we divided the dataset into five smaller datasets, and five models were constructed on each of the five different dataset patterns (Figure 1). For verification, we performed a PCA of each dataset to ensure unbiased segregation using 11 representative descriptors that are generally considered important for synthetic expansion.^28^ As shown in Figure 2, the separation was well balanced and the cumulative contribution ratio of PC1, PC2, and PC3 explained 82.3%.

Kosugi and Hosea^24^ constructed a prediction model using in-house compounds as a regression model for rat CL. Their models used 330 molecular descriptors and eight algorithms including random forest and radial basis functions. However, the prediction performance was not sufficient because only conventional ML algorithms were used. In this study, we constructed rat regression models using molecular descriptors obtained from the molecular operating environment (MOE), alvaDesc, and ADMET Predictor software and DataRobot, which can simultaneously verify multiple algorithms. The evaluation metrics showed that *R*^2^ and RMSE were 0.625–0.669 (average 0.649) and 0.295–0.318 (average 0.306) as shown in Table 1 and Figure S1. Although a direct comparison is difficult because of the different compounds used, our models performed better than previous models because we used multiple software to calculate molecular descriptors and multiple algorithms.

In attempts to improve the prediction performance, multiple QSAR models (ensemble model, consensus model, combinatorial QSAR, and models that use new hybrid descriptors) that use multiple features and multiple algorithms have been reported.^31−36^ Kim et al.^33^ and Zhang et al.^36^ used multiple descriptor software such as MOE, Dragon, and MolConnZ and multiple algorithms such as random forest, support vector machine, and *k*-nearest neighbor and proposed prediction models that used the mean values in these models. All of these QSAR models use molecular descriptors as compound features and generate different molecular descriptors using multiple software. Instead, our model uses compound features as molecular descriptors and images and combines them to obtain a higher prediction performance than conventional ML.^21^ In addition to using the features of compounds, Kim et al.^33^ and Wang et al.^37^ used biological descriptors related to membrane transporters and constructed a prediction model which improved the prediction performance. Kosugi and Hosea^24^ improved the prediction performance of their model using in vitro experimental values as a new feature in addition to molecular descriptors. These findings indicated that the inclusion of different types of features might improve the prediction performance of QSAR models. Therefore, in this study, the prediction probability, which is the continuous value obtained from DeepSnap-DL, was used as a new feature for constructing our regression model. The combination model that used the prediction probability of DeepSnap-DL and molecular descriptors improved the evaluation metrics [from *R*^2^ = 0.625–0.669 (average is 0.649) to *R*^2^ = 0.710–0.769 (average is 0.736) and from RMSE = 0.295–0.318 (average is 0.306) to RMSE = 0.247–0.278 (average is 0.265)] as shown in Table 1 and Figures S1 and S2. Similar results were obtained when different random seeds were used as shown in Table S2. To avoid the risk of overfitting and to keep the number of descriptors below 1/10 of the training datasets (1236 compounds), we constructed models by selecting the top 100 descriptors based on importance. The results of the prediction model with the number of descriptors restricted to 100 using DataRobot are shown in Tables S3 and S4. Even with the reduced number of descriptors (100), the prediction performance of the combination model using the prediction probability of DeepSnap-DL was better than the performances of molecular descriptor-based methods (from *R*^2^ = 0.536–0.625 to *R*^2^ = 0.696–0.763 and from RMSE = 0.317–0.350 to RMSE = 0.250–0.287). Although it is difficult to make direct comparisons between different compounds, the combination model showed a higher performance than the rat CL prediction model (*R*^2^ = 0.555, RMSE = 0.332) reported by Kosugi and Hosea.^24^ The relationship between the prediction probability of DeepSnap-DL and rat CL is shown in Figure S3. The prediction probability of DeepSnap-DL tended to increase as the rat CL value increased (*R*^2^ = 0.359). Permutation importance was used to calculate the effective features for the combination model. The average effect of the prediction probability of DeepSnap-DL was the highest among the descriptors (Table 3), and it was also the highest when only 100 descriptors were used (Table S5). These results indicate that the prediction probability of DeepSnap-DL is an important descriptor in both prediction models. Thus, the prediction probability of DeepSnap-DL was the most powerful descriptor for rat CL prediction, indicating why the combination model showed improved performance.

The descriptors nFCharge+, which is related to charge, and BCUT_SLOGP_0, which is related to lipophilicity, were selected as important descriptions in the molecular descriptor-based methods (Table 2). In the combination model, BCUT_SLOGP_0 was selected as the second most important descriptor after prediction probability of DeepSnap-DL (Table 3). Thus, molecular descriptors related to charge and lipophilicity were considered to be important in constructing the rat CL prediction model. In the prediction model for rat CL constructed by Kosugi and Hosea,^24^ the same lipophilicity and charge-related descriptors were used. Molecular descriptors related to lipophilicity and charge/ionic state were also used in human CL prediction models after variable selection, although the species are different.^5,38^ CL involves multiple mechanisms, including membrane permeability, metabolism, transporters, and protein binding,^22,39^ and the CL excretion route can be classified as metabolism, renal excretion, and biliary excretion.^39^ It is reported that membrane permeability, metabolism, transporter, and protein binding are related to lipophilicity and the ionic state.^40,41^ Therefore, it is assumed that CL is related to lipophilicity and the ionic state. Varma et al.^42^ proposed the extended clearance classification system (ECCS), which is a detailed classification of the CL excretion route based on membrane permeability, molecular weight, and the ionic state of the compounds. The ECCS classifies compounds as metabolism, renal excretion, and hepatic uptake based on their excretion route. Lipophilicity and ionic state are relevant to the CL excretion route because membrane permeability is related to lipophilicity, and the ionic state of compounds are considered in ECCS. Other studies have related CL values to lipophilicity and the ionic state.^40,41^ From these findings, the selection of lipophilicity and the ionic state as important molecular descriptors in our prediction model was reasonable. Interestingly, descriptors related to lipophilicity and charge/ionic state were highly ranked even when the prediction probability of DeepSnap-DL was included (Tables 3 and S5). This finding suggests that the predication probability of DeepSnap-DL may be a new descriptor independent of lipophilicity and charge/ionic state.

To investigate the possibility of applying the method to other parameters, we evaluated molecular descriptor-based methods and the combination model using the estimating the aqueous solubility (ESOL) dataset from Wu et al.^25^ To calculate the prediction probability of DeepSnap-DL, we constructed classification models for ESOL. The constructed models showed high prediction performance with AUCs of 0.962–0.966 (Table S6). Prediction models were also constructed using 100 descriptors that were selected based on their importance for the regression model. As shown in Table S7, the molecular descriptor-based methods had average *R*^2^ and RMSE values of 0.943 and 0.502, respectively. Although a direct comparison is difficult because the split datasets were different, the best RMSE score reported by Wu et al. was 0.58.^25^ Therefore, we successfully constructed models that produced high prediction performances using multiple software and algorithms. Furthermore, the combination model was constructed by adding the prediction probability of DeepSnap-DL obtained by the classification model. The average *R*^2^ and RMSE values for the combination model were 0.950 and 0.468, which is an improvement in the prediction performance compared with the prediction performances of molecular descriptor-based methods. Similar to rat CL, the prediction probability of DeepSnap-DL was the most important descriptor used in the prediction model (Table S8). These results indicate that the combination model was useful not only for rat CL but also for ESOL. However, it is not guaranteed to be useful in predicting all targets. Physiological phenomena with complex pathogenic mechanisms, especially toxicity represented by LD_50_ (Lethal Dose 50), may be challenging targets. Identification of the effective target type for the combination model remains an important issue in future.

## Conclusions

In this study, we constructed a novel high-performance regression model of rat CL by combining the features of compounds as images and molecular descriptors. This approach may be effective not only for rat CL and ESOL but also for other regression models of, for example, pharmacokinetic, toxicity, and pharmacological activity parameters. Therefore, this approach may be useful when the performance of conventional ML is low. Because this approach is based on the structures of compounds, it is expected to be widely applicable in various fields related to compounds.

## Methods

### Experimental Data

All the animal experimental procedures were approved by the Animal Ethics Committee of Japan Tobacco Inc, Central Pharmaceutical Research Institute.

The rat CL dataset used in this study contained the same 1545 in-house compounds used previously.^21^ Briefly, the CL data for all these compounds were calculated by noncompartmental analysis based on their plasma concentrations after intravenous administration in rats. In the classification model, the threshold was set to 1 L/h/kg, as reported previously.^21^ In the regression model, the CL value of each compound was transformed to log(CL), as reported by Kosugi and Hosea.^24^

The ESOL dataset (1128 compounds) was obtained from Wu et al.^25^ The threshold value was set at −3 in the classification model.

### Calculation of Molecular Descriptors

In this study, we used the 4795 descriptors of 1545 compounds that we had used previously for rat CL.^21^ Furthermore, 6482 descriptors of 1128 compounds were used for the ESOL dataset. Briefly, all the compounds were optimized using “Rebuild 3D”, and force field calculations (amber-10: EHT) were conducted in MOE version 2019.0102 (MOLSIS Inc., Tokyo, Japan). The resulting three-dimensional (3D) structures were saved in SDF format. Molecular descriptors were calculated using MOE, alvaDesc (1.0.16 for rat CL and 2.0.2 for ESOL) (Alvascience srl, Lecco, Italy), and ADMET Predictor (9.5.0.16) (Simulations Plus, Lancaster, CA, USA). String-type descriptors from ADMET Predictor and descriptors with variance 0 from alvaDesc were removed for rat CL. String-type descriptors were removed and the modeling descriptor was used on ADMET Predictor for ESOL. LogS and h_logS from MOE, and ESOL from alvaDesc were removed because of the risk of data leakage.

### Chemical Space Analysis for the Five Separated Datasets

After applying stratified random sampling, the 1545 compounds in the dataset were separated randomly into five datasets as shown in Figure 1. Training (1236 compounds) and test (309 compounds) datasets were selected by combining the five datasets and verified using five split patterns (Figure 1). To examine the chemical space of each of the five datasets, a PCA was performed using JMP Pro software 14.3.0 (SAS Institute Inc., Cary, NC, USA) and 11 descriptors, namely, molecular weight, SlogP (log octanol/water partition coefficient), TPSA (topological polar surface area), *h*_log *D* (octanol/water distribution coefficient [pH 7]), *h*_p*K*_a_ (acidity [pH 7]), *h*_p*K*_b_ (basicity [pH 7]), a_acc (number of H-bond acceptor atoms), a_don (number of H-bond donor atoms), a_aro (number of aromatic atoms), b_ar (number of aromatic bonds), and b_rotN (number of rotatable bonds) as previously reported.^21,28^ The first three principal components (PC1–PC3) were calculated.

### DeepSnap

Using the SDF files prepared by the MOE application, the chemical structures of the compounds were depicted as 3D ball-and-stick models in which different colors represent different atom types using Jmol, an open-source Java viewer software for 3D molecular modeling of chemical structures, as previously reported.^14−20^ The 3D chemical structures were captured automatically as snapshots with user-defined angle increments for the *x*, *y*, and *z* axes. Accordingly, the 3D chemical structure was gradually rotated 360° on each axis and snapshots were captured from all viewing directions. In a previous study, we used images generated from four angle increments (65°, 65°, 65°) (85°, 85°, 85°) (105°, 105°, 105°), and (145°, 145°, 145°) for the *x*, *y*, and *z* axes to compare the results.^21^ On the basis of the previous results, we selected the angle (145°, 145°, 145°) and obtained 27 images per compound. These images were used as features to construct the prediction model. Similarly, images of the compounds for ESOL were also obtained at (145°, 145°, 145°). Other parameters were selected based on previous studies^14−21^ as follows: image pixel size: 256 × 256; molecule number per SDF file to split into: 100; zoom factor (%): 100; atom size for van der Waals radius (%): 23; bond radius (mÅ): 15; minimum bond distance: 0.4; and bond tolerance: 0.8. The snapshot images were saved as 256 × 256-pixel resolution PNG files (RGB). These compounds images were divided into three types of datasets, DeepSnap (training), DeepSnap (validation), and test, as described in Figure 1. After applying stratified random sampling, the training datasets were separated into DeepSnap (training) and DeepSnap (validation) at a ratio of 3:1 for each dataset pattern (Figure 1). For the ESOL dataset, stratified random sampling was applied, and then, the datasets were separated into DeepSnap (training), DeepSnap (validation), and test sets at a ratio of 8:1:1 in accordance with the ratios reported previously.^25^

### DeepSnap-DL for the Classification Model

All PNG images produced by DeepSnap were resized using the NVIDIA DL GPU Training System (DIGITS) version 6.0.0 (NVIDIA, Santa Clara, CA, USA), on four-GPU Tesla-V100 (32 GB) systems, with a resolution of 256 × 256 pixels as input data, as previously reported.^14−21^ We used a pre-trained open-source DL model, Caffe,^43^ and the open-source software on a Ubuntu 16.04 LTS for rapid training and fine-tuning. The deep convolutional neural network architecture used in this study was GoogLeNet,^44^ and Adam^45^ was used for optimization. The prediction models were constructed with the DeepSnap (training) datasets using 300 epochs with a one snapshot interval and one validation interval in each epoch, one random seed, a learning rate of 0.000001, and a default batch size in DIGITS, as previously reported.^21^ The lowest loss value in the DeepSnap (validation) datasets, which is the error rate between the results obtained from the DeepSnap (validation) datasets and the corresponding labeled dataset, was chosen for the subsequent evaluation of prediction probabilities. The representative prediction probabilities for each image of one compound were calculated based on the medians of values captured at different angles for the *x*, *y*, and *z* axes as previously reported.^14−21^

### Construction of Regression Models Based on Molecular Descriptors (Molecular Descriptor-Based Methods) or Prediction Probability of DeepSnap-DL and Molecular Descriptors (Combination Model)

Model constructions based on 4795 molecular descriptors or prediction probability of DeepSnap-DL combined with 4795 molecular descriptors were performed using DataRobot (SaaS, DataRobot, Tokyo, Japan) for rat CL. For the ESOL dataset, prediction models were constructed using 6482 descriptors or using 6482 descriptors and the prediction probability of DeepSnap-DL. All the analyses were conducted from 18 May to 9 December 2021. DataRobot automatically performs a modeling competition in which a wide selection of algorithm and data preprocessing techniques compete with one another.^29,30^ Various preprocessing methods were automatically applied to the data. For numerical values, standardization and imputing missing values were used.^29,30^ At the start of the modeling, 20% of the training dataset was randomly selected as the hold-out test dataset and excluded from the training run (step 1, Figure 1). Fivefold cross-validation was used, and the partitions were determined with stratified sampling (Figure 1). Models were created using the automated ML platform DataRobot. Over 30 models were created, including “ensemble models” that used several ML algorithms. Multiple ensemble models were generated; single ML models with different algorithms (e.g., XGBoost, random forest, support vector machines, neural networks, and regularized regression models such as elastic net) were combined. The ensemble models also applied various methods such as average and generalized linear models. The 4795 molecular descriptors were reduced to 4331–4338 descriptors by DataRobot (step 1, Figure 1) for rat CL prediction. After competition of each algorithm run using these descriptors, algorithms were selected as the final algorithm based on the RMSE of the validation results. Then, the best model was constructed using all the training data (80%) and this final model was used to calculate the evaluation metrics of the test datasets (step 2, Figure 1). For the ESOL dataset, 100 descriptors were selected according to their importance to construct the final prediction model. The representative effect of feature importance was calculated based on the permutation importance and by averaging each value on the five models.

### Evaluation of the Prediction Models

The performance of each model in predicting the rat CL was evaluated based on *R*^2^ and RMSE calculated using KNIME (4.1.4), an open-source data mining platform (KNIME, Konstanz, Germany). RMSE was defined as
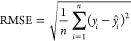
where *y*_*i*_, , and *n* are actual values, predicted value, and the size of the dataset, respectively.

## Supporting Information Available

Scatter plots of external test datasets using molecular descriptors; and using molecular descriptors and prediction probability of DeepSnap-DL; and relationship between rat CL and DeepSnap-DL probability (PDF)
Final algorithms selected by DataRobot using 4331–4338 descriptors; results with different random number seeds; final algorithm; internal validation and external test results using 100 molecular descriptors; 100 molecular descriptors used in the rat CL prediction models using molecular descriptors and prediction probability of DeepSnap-DL; results of the classification for ESOL; external test results; and 100 molecular descriptors used in the ESOL models using molecular descriptors and prediction probability of DeepSnap-DL (XLSX)

## Abbreviations

QSAR: Quantitative structure–activity relationships
ADMET: absorption, distribution, metabolism, excretion, and toxicity
ML: machine learning
DL: deep learning
DeepSnap-DL: DeepSnap and Deep Learning
CL: clearance
AUC: area under the curve
*R*^2^: correlation coefficient
RMSE: root mean square error
PCA: principal component analysis
PC: principal component
MOE: molecular operating environment
ECCS: extended clearance classification system
ESOL: estimating the aqueous solubility
3D: three-dimensional
DIGITS: the NVIDIA DL GPU Training System
LD_50_: Lethal Dose 50
