# Supplementary material for: Novel QSAR Approach for a Regression Model of Clearance That Combines DeepSnap-Deep Learning and Conventional Machine Learning
Source: ACS Omega. 2022 May 11;7(20):17055–62. doi: 10.1021/acsomega.2c00261 (PMC9134387; doi:10.1021/acsomega.2c00261)
Supplement: Supplementary file 1 — ao2c00261_si_001.pdf [file ao2c00261_si_001.pdf]

Novel QSAR approach for regression model of clearance that combines DeepSnap-Deep Learning and conventional machine learning

Hideaki Mamada[1,2], Yukihiro Nomura[2], Yoshihiro Uesawa[1]*

[1]Department of Medical Molecular Informatics, Meiji Pharmaceutical University, 2-522-1, Noshio, Kiyose, Tokyo 204-8588, Japan

[2]Drug Metabolism and Pharmacokinetics Research Laboratories, Central Pharmaceutical Research Institute, Japan Tobacco Inc., 1-1, Murasaki-cho, Takatsuki, Osaka 569-1125, Japan.

[*]Corresponding author.

Tel.: +81-42-495-8983; Fax: +81-42-495-8983;

E-mail address: uesawa@my-pharm.ac.jp (Y.U.)

Supporting Information 1

Supporting Tables (file type: Excel)

Table S1. Final algorithm selected by DataRobot using 4331–4338 descriptors.

Table S2. Internal validation and external test results using 4331-4338 descriptors with different random number seeds.

Table S3. Final algorithm selected by DataRobot using 100 descriptors

Table S4. Internal validation and external test results using 100 descriptors with different random number seeds.

Table S5. One hundred descriptors in rat CL prediction models using molecular descriptor and prediction probability of DeepSnap-DL

Table S6. Prediction performances for aqueous solubility using DeepSnap-Deep Learning

Table S7. External test results for aqueous solubility using molecular descriptor and prediction probability of DeepSnap-DL with different random number seeds.

Table S8. One hundred descriptors in for aqueous solubility prediction models using molecular descriptor and prediction probability of DeepSnap-DL

Supporting Information 2

Supporting Figures (file type; WORD)

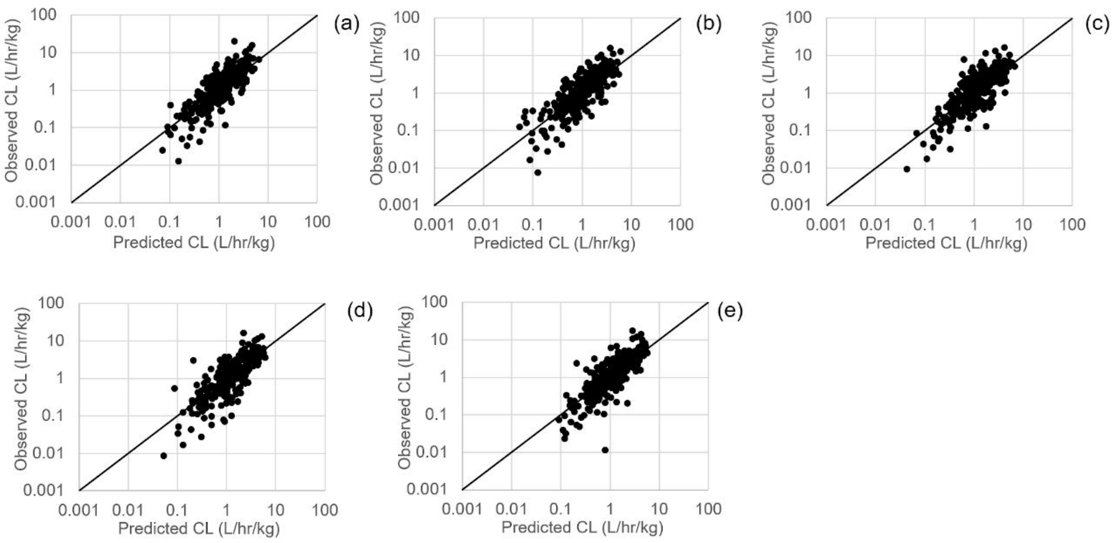

Figure S1. Scatter plots of external test sets using molecular descriptors

(a) Pattern1, (b) Pattern2, (c) Pattern3, (d) Pattern4, and (e) Pattern5. Each dot indicates a compound in the test set (n = 309); the solid line indicates unity. All results are shown for seed=1.

CL, clearance.

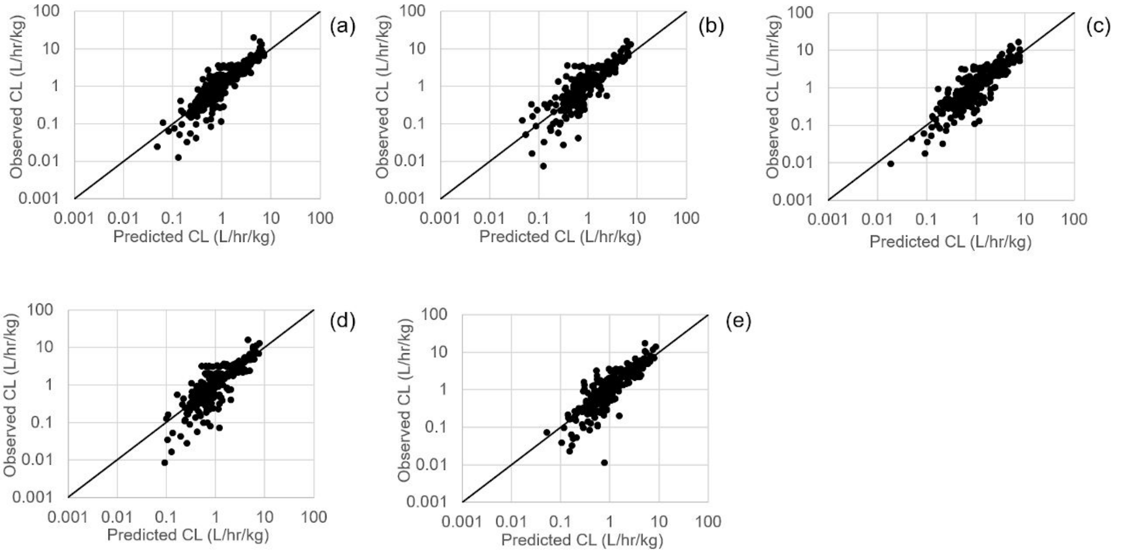

Figure S2. Scatter plots of external test sets using molecular descriptors and prediction probability of DeepSnap-DL

(a) Pattern1, (b) Pattern2, (c) Pattern3, (d) Pattern4, and (e) Pattern5. Each dot indicates a compound in the test set (n = 309); the solid line indicates unity. All results are shown for seed=1.

CL, clearance; DeepSnap-DL, DeepSnap and Deep Learning.

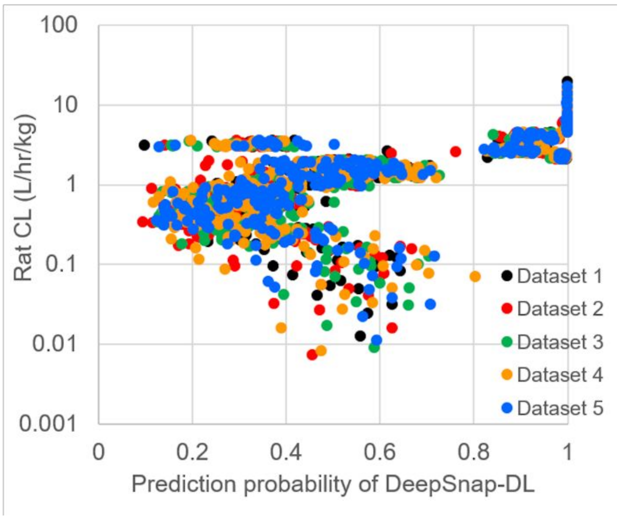

Figure S3. Relationship between rat clearance and prediction probability of DeepSnap-DL

The five colors show the results of the test compounds (n = 5 × 309) in each dataset pattern for seed (=1).

CL, clearance; DeepSnap-DL, DeepSnap and Deep Learning.
